# An Empirical Test of the Concept of the Adaptively Intelligent Attitude

**DOI:** 10.3390/jintelligence12050049

**Published:** 2024-04-30

**Authors:** Robert J. Sternberg, Arezoo Soleimani Dashtaki, Banu Baydil

**Affiliations:** 1 Department of Psychology, College of Human Ecology, Cornell University, Ithaca, NY 14853, USA; asc329@cornell.edu; 2 Department of Statistics, Arts and Sciences, Columbia University, New York, NY 10027, USA; banu.baydil@columbia.edu

**Keywords:** adaptation, intelligence, intelligent attitude, dispositions, adaptive intelligence, fluid intelligence

## Abstract

This study provides an empirical test of a previously proposed assertion that intelligence as adaptation has an attitudinal as well as an ability component. The ability component deals with what the basic knowledge and skills are that underlie intelligence, and how much of each one an individual has. The attitudinal component deals with how an individual chooses to deploy the abilities they have. In other words, to what use are the abilities put? It is argued that it is impossible fully to separate the measurement of the ability component from the attitudinal one. In a diverse population, even taking an intelligence test will show itself to involve an attitude toward the test, which may enhance or detract from performance, as when one sees the test as irrelevant or harmful to one’s life, or as a sociocultural misfit to one’s life experience. To succeed, people need not only to have abilities, but attitudes that put those abilities to effective use to accomplish individuals’ life goals. In the study, we found that intelligent attitudes are related, but non-identical, to germane constructs, such as wisdom, the need for cognition, creativity, and openness to experience. Scores on the attitudinal measure were not related to scores on tests of fluid intelligence and academic abilities/achievement. Thus, the range of attitudes regarding how to deploy intelligence can vary over ability levels.

## 1. Introduction

On the one hand, scientists and society alike typically think of intelligence as an ability or a set of abilities (e.g., Carroll 1993; Deary 2020; Kaufman et al. 2020; McGrew 2009; Spearman 1904; Sternberg 2020). On the other hand, both scientists and laypeople know that people who have high intelligence often act in surprisingly “stupid” or foolish ways (Aczel 2020; Sternberg 2002). In some cases, they may lack emotional intelligence (Rivers et al. 2020), social intelligence (Kihlstrom and Cantor 2020), cultural intelligence (Ang et al. 2020), or what Gardner calls interpersonal intelligence (Gardner 2011); but in other cases, the problem may seem to be not a lack of ability but rather the attitude with which they approach a task requiring intelligence: they seem to self-sabotage their performance by going into the task with an attitude that will lead to failure or defeat; Sternberg (2022) has referred to this phenomenon as a failure of intelligent attitude. Relevant also is the construct of self-handicapping, whereby people purposely set up obstacles in their way, sometimes to blame failure in tasks on external causes rather than on themselves (e.g., Berglas and Jones 1978; Jones and Berglas 1978).

At one point in the history of social psychology, investigators recognized how seeking cognitive consistency was a major motivating source in people’s lives (e.g., Abelson et al. 1968; Brehm and Cohen 1962; Cialdini et al. 1981). People could deal with cognitive inconsistencies in more or less adaptive (what are here called “adaptively intelligent”) ways. For example, if someone finds out negative information about a person in power or a person seeking power, or a loved one, for that matter, they can, in Piaget’s (1952) terminology, assimilate the information and accept it, or accommodate that negative knowledge, creating a new cognitive structure; or if accommodation fails, they can simply deny the validity of the information or even that the information exists.

In this article, an ability is defined as a developed cognitive or related capacity that can be modified, at least to some degree, through instruction and effort (Sternberg 2022). This definition is largely consistent with dictionary definitions, such as “the physical or mental power or skill needed to do something” (Cambridge Dictionary, https://dictionary.cambridge.org/us/dictionary/english/ability, accessed 16 April 2024) or “developed skill, competence, or power to do something, especially (in psychology) existing capacity to perform some function, whether physical, mental, or a combination of the two, without further education or training, contrasted with capacity, which is latent ability” (*Oxford Reference*, https://www.oxfordreference.com/search?q=ability&searchBtn=Search&isQuickSearch=true, accessed 16 April 2024). None of the definitions imply innateness or immutability. Other related definitions can be found in Cambridge handbooks on abilities (e.g., Kaufman and Sternberg 2019; Sternberg 2020).

In contrast, an attitude is defined as a developed mindset or approach toward something that is capable of and susceptible to change (Sternberg 2022). This definition, again, is similar to other standard definitions, such as: “a feeling or opinion about something or someone, or a way of behaving …” (Cambridge Dictionary, https://dictionary.cambridge.org/us/dictionary/english/attitude, accessed 16 April 2024), or “the way in which a person views and evaluates something or someone (Oxford Reference, https://www.oxfordreference.com/display/10.1093/oi/authority.20110803095433168, accessed 16 April 2024). Our definition, again, emphasizes the possibility of modifiability. Other related definitions can be found in the literature on attitudes (e.g., Banaji and Heiphetz 2010; Rajecki 1990; Zanna and Rempel 2008).

From this vantage point, an individual can have an ability, but without the attitude to deploy that ability effectively, or even at all. In some cases, reckless attitudes, such as toward gambling one’s money without setting adequate limits, may undermine the utilization of one’s cognitive abilities. Thus, the ability may remain latent and hence underutilized, misutilized, or even unutilized.

The argument underlying this article is that the deployment of intelligence always requires both intelligence as an ability and intelligence as an attitude. People sometimes have the ability but decide not to use it. Or they may not have so much ability, but they have the attitude to use what ability they have effectively.

Intelligence as an attitude is related to other cognitive, personality, and motivational characteristics, which are discussed in some detail in Sternberg (2022). For example, a related construct is the need for cognition (Cacioppo and Petty 1982; Cacioppo et al. 1996), but the constructs are not the same. The construct under examination is a basis for whether or how one deploys one’s intelligence, not an underlying need for cognitive functioning in general.

It might seem that how one deploys one’s intelligence is an issue entirely separate from the level of one’s intelligence. At a theoretical level, it is separate. But at a practical level, the level of intelligence can never be fully separated from the deployment of intelligence because performances on the cognitive or other tests used to measure intelligence all represent deployments of intelligence, not pure indicators of intelligence independent of deployment. When one takes a test, one is deploying one’s intelligence. Some people might not care about how they perform on a test, and so do poorly on it. Others might care but not understand the tacit knowledge of test-taking—so-called “test-wiseness”—and so do poorly on the tests. Still others might be very effective in deploying their intelligence, just not on the kinds of conventional tests used to measure intelligence in the West (see, e.g., Cole 1998; Luria 1976; Sternberg and Preiss 2022). In highly collectivist cultures, the very act of taking a test individually may seem irregular (Greenfield 1997). And reaction-time tests, or other timed tests, in cultures that view intelligence as comprising slow, deep thought rather than quick, superficial thought, might seem to be counter to what they believe intelligence to be (Sternberg 2004). In other words, attitudes always mediate any expression of intelligence, including on tests designed to measure intelligence as an ability. When one measures intelligence, an attitude toward the testing process becomes “baked” into the score.

The view of an attitudinal component to intelligence was first proposed by Sternberg (2022), but the original proposal did not contain a measure to assess intelligent attitudes. The purpose of this article is to present such a measure and to report on data relevant to validating the measure. This article does not represent a full construct validation, but rather a start toward understanding convergent and discriminant relations between adaptively intelligent attitudes and related constructs. The current research is best viewed as a prologue to a construct validation rather than as a construct validation in itself. There simply is not enough theoretical or empirical work yet to undertake a serious construct validation of attitudinal aspects of intelligence.

The target of our investigation here is what Sternberg (2021) has referred to as *adaptive intelligence*, which is intelligence as it is involved in adaptation to the environment, a key component of most definitions of intelligence (e.g., Gottfredson 1997; “Intelligence and Its Measurement” 1921). Sternberg (2019) introduced the concept of *adaptive intelligence* in this journal, and defined it as “*intelligence that is used in order to serve the purpose of biological adaptation, which, for humans, always occurs in, and hence is mediated by, a cultural context”* (p. 2). The attitudes measured are those attitudes required for adaptation to the world, which is what we believe intelligence is for. Those attitudes, according to the theory, relate to creative, analytical, practical, wise, and meta-intelligent thinking (where meta-intelligent thinking is the choice to utilize creative, analytical, practical, or wise thinking, according to the circumstances—Sternberg et al. 2021). Intelligent attitudes are not necessarily specific to any particular situations, such as taking an intelligence test or one of its proxies or performing well in a course in school.

Adaptive intelligence can be measured (Sternberg et al., forthcoming). Examples are given in Sternberg (2021). An example would be knowing how to avoid getting infected by an illness that can rather easily be prevented or knowing how to treat an illness, to the extent possible, so that one works toward getting better (see, e.g., Sternberg 2021).

Attitudes toward adaptive intelligence are key to its deployment: some people do not really care all that much about how they adapt to the environment but may care about other things, for example, making money at any cost or taking illegal drugs, which they know are maladaptive and potentially toxic.

Adaptive intelligence is always a person × task × situation interaction (Sternberg 2021). How well a person adapts will depend upon the tasks they choose or are chosen to confront (e.g., engineering problems versus writing a literary analysis) and the kinds of environmental contexts in which they confront them (e.g., an environment in which one can say what one wants versus an environment in which one can expect to be imprisoned if one says or writes something of which one’s government disapproves). As Bronfenbrenner and Ceci (1994) have pointed out, there are always environmental forces that create proximal processes that modify how intelligence and other cognitive abilities develop and manifest themselves.

As an example of effects of proximal processes, countries can degenerate quickly as a result of contextual forces. Criminals can become presidents, prime ministers, and dictators, simply because people do not want to believe the facts that are staring them in the face. One would expect, therefore, that dogmatism and authoritarianism would relate negatively to adaptively intelligent attitudes, whereas openness to experience, need for cognition (to process novel and sometimes unpleasant information), critical thinking, and wisdom to consider information in a balanced way would relate positively to adaptively intelligent attitudes.

Interestingly, perhaps, intelligence as an ability will not necessarily show much, if any, correlation with intelligence as an attitude. Abilities and attitudes are simply different things: the knowledge and skills one possesses, and how one deploys them, are different issues. Oddly, the individual who perhaps best pointed this out was Sir Arthur Conan Doyle. In Doyle’s (2003) Sherlock Holmes series of detective stories, Sherlock Holmes had extremely well-developed knowledge and ability for criminological investigation—unusual perceptive, inductive, and deductive reasoning abilities—and in his fictional world, he was an amazing detective. His brother Mycroft Holmes, introduced, for example, in *The Greek Interpreter*, was described as considerably more capable than Sherlock Holmes, but did almost nothing with his abilities, preferring to sit around in the Diogenes Club of London, a club for asocial men, each of whom wanted to have nothing to do with the others. Sherlock, in other words, had a profusion of intelligent attitudes, Mycroft, not so many. Perhaps relevant is that Doyle himself, brilliant though he was, was also a spiritualist who had strange beliefs, such as in people’s ability to communicate with the dead. One does not necessarily show in one’s life the abilities and attitudes about which one writes.

Thus, we expected our measure of attitudes relevant to adaptive intelligence to be positively correlated with dispositions and characteristics relevant to the deployment of intelligence—for example, the need for cognition, openness to experience, and wisdom (measured as a disposition). We expected our measure to be negatively correlated with dispositions that tend to close off adaptive intellectual functioning—for example, authoritarianism and dogmatism. And, we expected little or no correlation of our measures with *levels* of intelligence, as measured by fluid intelligence tests and proxy measures of intelligence (ACT, SAT, two tests used for college admissions in the United States).

## 2. Method

### 2.1. Participants

A total of 197 undergraduate students at a selective university in the Northeastern United States participated in the data collection. Of these, 10 participants were eliminated because of missing values in adaptive intelligence (AIAS) items. Of the remaining total of 187 participants, 145 were female, 41 were male, and 1 of them refused to identify their sex (i.e., they chose, “Prefer not to say”). The average age was 20.14 years, with a standard deviation of 1.29 years. The distribution of self-reported ethnicities was 34.9% Asian and Asian American, 36.9% White or Caucasian, 6.4% Black or African American, 12.3% Hispanic or Latino, 0.5% American Indian or Alaska Native, and 7% of two or more races; 1.6% preferred not to reveal their ethnicity. Two participants were eliminated for providing nonsensical answers (GPAs over 4.3, given that the maximum possible GPA at the institution where the study was conducted is 4.3).

### 2.2. Materials

There was a total of 11 assessments presented in the form of an online survey, administered through Qualtrics. These assessments consisted of (1) an Adaptive Intelligence Attitudes Scale (AIAS) we have created, composed of 37 items; (2) two psychometric assessments, which included Letter Sets and Figure Classification in order to assess fluid intelligence (from the *Kit of Factor-Referenced Cognitive Tests*; Ekstrom et al. 1976); (3) the Very Short Authoritarianism (VSA) scale (Bizumic and Duckitt 2018); (4) the 12-item Three-Dimensional Wisdom Scale (Thomas et al. 2017); (5) the Creativity Scale from Seligman et al. (2004) Values in Action (VIA); (6) the Critical Thinking Dispositions Scale (CTDS) (Sosu 2013a, 2013b); (7) the Need for Cognition (NFC) scale (Cacioppo et al. 1984); (8) an Openness to Experience (OE) scale (IPIP-NEO-60 2017) (Maples-Keller et al. 2019); (9) the Dogmatism (DOG) scale (Altemeyer 2002); (10) the Balanced Inventory of Desirable Responding—Short Form (BIDR-16) (Hart et al. 2015); and (11) a demographic questionnaire we constructed.

For developing and validating the Adaptive Intelligence Attitudes Scale, steps were taken based on Fenn et al.’s (2020) guidelines. The theoretical framework was that of adaptive intelligence as an ability and as a set of attitudes relevant to adaptive intelligence (Sternberg 2021, 2022). We further considered the role of what Sternberg and his colleagues have called meta-intelligence (Sternberg et al. 2021), which is the executive integration of analytical intelligence, practical intelligence, creativity, and wisdom. Because these constructs overlap—in the theory of adaptive intelligence, all are controlled by metacomponents (executive processes)—items do not necessarily clearly fall into one category or another. That is, a given item might measure more than one type of attitude, for example, both intelligent and wise attitudes.

Adaptive Intelligence Attitudes: the Adaptive Intelligence Attitudes Scale. In this study, intelligent attitudes were measured by 40 items (37 items measuring adaptive intelligence attitudes and 3 lie items) from a newly constructed Adaptive Intelligence Attitudes Scale (AIAS). Participants provided their responses on a scale ranging from 1 (never) to 5 (almost always or always). The items are listed in Appendix A.

Fluid Intelligence: Letter Sets and Figure Classification tests. In this study, Letter Sets and Figure Classification served as the two assessments used for measuring fluid intelligence. The Letter Sets test required participants to rule out one letter set that did not fit in with the four other letter sets given. The Figure Classification test required participants to select and categorize each given figure into a group based on feature similarity. The tests were taken from the *Kit of Factor-Referenced Cognitive Tests* (Ekstrom et al. 1976), tests originally developed at the Educational Testing Service (ETS), which have been used extensively by us and others in the assessment of cognitive abilities. This section was scored based on how many correct answers were given. Each correct answer yielded one point.

Authoritarianism: the Very Short Authoritarianism (VSA) scale. Authoritarianism was assessed by the six-item Very Short Authoritarianism scale (Bizumic and Duckitt 2018). This is a shortened version of Altemeyer’s (1996) authoritarianism scale. Items are classified into three subdimensions—Authoritarian Aggression, Conservatism, and Traditionalism. Participants provided their responses on a Likert scale, ranging from −4 (strongly disagree) to +4 (strongly agree). A sample item is, “What our country needs most is discipline, with everyone following our leaders in unity.” Bizumic and Duckitt (2018) found the alpha reliability coefficient for the VSA scale to be 0.78 in a UK sample, with a mean inter-item correlation of 0.38, and 0.71 in a US sample, with a mean inter-item correlation of 0.29.

Creativity: Values in Action (VIA). In the study, to measure creativity, Seligman et al.’s (2004) Values in Action (VIA) scale was used. This self-report tool measures 24 character strengths using a five-point Likert scale (1 = extremely uncharacteristic, to 5 = extremely characteristic) to measure how frequently one perceives oneself as exhibiting certain behaviors. Among them, 8 items measure creativity. This measure was found to have acceptable internal consistency reliability (all alphas > 0.70) and temporal reliability (test/retest > 0.70) for the overall test, and an alpha of 0.85 for creativity strength (Seligman et al. 2004).

Wisdom: the Three-Dimensional Wisdom Scale. In this study, the 12-item Three-Dimensional Wisdom Scale (3D-WS-12) (Thomas et al. 2017), with cognitive, reflective, and affective (compassionate) subscales, was used. This 12-item measure utilizes five ordered categorical response options (1 = “strongly agree” or “definitely true of myself” through 5 = “strongly disagree” or “not true of myself”). A sample item is “I’m easily irritated by people who argue with me.” Thomas and his colleagues (2017) found the alpha coefficient for all items to be 0.73. This scale is based on Ardelt’s (2003, 2004) conception of wisdom.

Critical thinking: the Critical Thinking Dispositions Scale. The Critical Thinking Dispositions Scale (CTDS) of Sosu (2013a) was used to measure critical thinking as a disposition. This is a self-report scale with 11 items that utilizes a 5-point Likert-type response scale (CTDS) (1 = strongly disagree, 5 = strongly agree) and two subscales—Critical Openness and Reflective Skepticism. An example of an item is “I often re-evaluate my experiences so that I can learn from them.” Sosu (2013b) examined the validity and reliability of the CTDS using multi-group confirmatory factor analysis (MGCFA). Results from this analysis show that the factor structure of the CTDS is equivalent across undergraduate and graduate groups and that participants in both groups understood the items in the same way. Sosu (2013b) performed two studies, and Cronbach’s alpha was 0.79 in the first study and 0.81 in the second one.

Need for cognition: the Need for Cognition scale. The Need for Cognition scale (NFC; Cacioppo et al. 1984) adopts a 5-point Likert scale, where 1 denotes “extremely uncharacteristic of me” and 5 denotes “extremely characteristic of me.” This scale has 18 items. “I find satisfaction in deliberating hard and for long hours” is an example of a statement on this scale. A higher score indicates a higher tendency to enjoy deeper thinking. The theta coefficient, which is a maximized Cronbach’s alpha coefficient (Helms et al. 2006), was +0.90 for the 18-item NCS.

Openness to Experience: the NEO-60. For measuring openness to experience, one aspect of the IPIP–NEO–60 (Maples-Keller et al. 2019), which is a 60-item measure of the FFM broad domains of personality created from the 300-item IPIP–NEO, was used. Mean inter-item correlations and alphas for all FFM measures are above 0.78. Cronbach’s alpha for Openness to Experience was 0.78 (Maples-Keller et al. 2019). The current study used okthe six facets of openness: Imagination, Artistic Interests, Emotionality, Adventurousness, Intellect, and Liberalism. Each facet was measured using 2 items. One sample item is “I avoid philosophical discussions.” Participants were asked to depict their level of agreement with each statement, on a scale of 1 to 5, with 1 representing a very inaccurate statement about oneself and 5 representing a very accurate statement.

Dogmatism: the DOG scale. The 20-item DOG scale (Altemeyer 2002) was used to measure dogmatism. An example item is: “The things I believe in are so completely true, I could never doubt them.” Participants indicated their agreement with each item on a nine-point scale (1 = “Strongly Disagree”, 9 = “Strongly Agree”). Higher scores indicate oogreater dogmatism. The DOG scale was selected to measure dogmatism because its validity and reliability have been demonstrated across multiple studies. Altemeyer (2002) found that the items had a Cronbach’s alpha of 0.90.

Social desirability: the Balanced Inventory of Desirable Responding. In the study, the Balanced Inventory of Desirable Responding—Short Form (BIDR-16) (Hart et al. 2015) was administered to assess socially desirable responsiveness. It includes two subscales; Self-Deceptive Enhancement (SDE), which refers to one’s belief in the truth of own positive self-image, and Impression Management (IM), which is a measure of one’s conscious attempts to favorably influence others’ views of one’s image. There are 16 items on a 7-point scale (1 = “Strongly Disagree”, 9 = “Strongly Agree”). “I sometimes tell lies” is one of the items. Hart et al. (2015) examined test–retest reliability of the scale over 2 weeks, which was r = 0.74 for IM and r = 0.79 for SDE.

Demographic questionnaire. The demographic questionnaire requested information about the participants, such as age, sex, ethnicity, year at the university, highest SAT and/or ACT score (if either exam was taken), and cumulative college GPA.

### 2.3. Design

The design of this study was correlational. The main dependent variable was the new Adaptive Intelligence Attitudes Scale. Other scores were used as independent variables to predict the dependent variable. Data were analyzed within subjects: every subject received every measure.

### 2.4. Procedure

The final version of the AIAS, along with other questionnaires, was provided to the participants in the form of an online survey through the Qualtrics platform. First, before taking the assessments, participants were asked to read and sign an informed consent form.

Upon signing consent, the participants proceeded to the two psychometric assessments: Letter Sets and then Figure Classification. The psychometric sections were automatically timed. Once the time limit was reached, the system forwarded each participant directly to the next section. The time limit for Letter Sets was 7 min and for Figure Classification it was 8 min. The following sections, including the Adaptive Intelligence Attitudes Scale, the Very Short Authoritarianism scale (Bizumic and Duckitt 2018), the 12-item Three-Dimensional Wisdom Scale (2017), the Creativity Scale (the Values in Action of Seligman et al. 2004), the Critical Thinking Dispositions Scale (2013), the Need for Cognition scale (1984), the Openness to Experience Scale (IPIP-NEO-60 2017), the Dogmatism scale (2002), the Balanced Inventory of Desirable Responding—Short Form (2015), and the demographic questionnaire, all did not have a time limit.

Upon completion of the study, a written debriefing form was presented to the participants.

## 3. Results

### 3.1. AIAS (Adaptive Intelligence Attitude Scale) Factor Analysis

In this section, we describe our internal validation of the AIAS.

Exploratory factor analysis was conducted to investigate the factor structure of the initially developed AIAS with 37 items and to refine the AIAS, if needed. Through a systematic iterative process, AIAS Items 4, 25, 14, 9, 20, 34, 17, and 26 were removed from the AIAS until a finalized robust factor structure was obtained. Details of AIAS factor analysis and refinement are presented in Section 3.1.4 Factor Analysis (Tables 3–5). The resulting AIAS had 28 items. Henceforth, the refined AIAS with 28 items is used in the analyses with other scales.

Results are summarized in a series of tables, as follows.

#### 3.1.1. Data Screening

The original dataset for AIAS factor analysis consisted of 37 AIAS items measured across 187 participants, after 10 cases with missing values were discarded via listwise deletion and 1 case with a higher Mahalanobis distance (*p* < 0.0001) (Mahalanobis distance function, faoutlier package (Chalmers and Flora 2015), R Statistical Computing Software Version 4.2.1 (23 June 2022 ucrt), henceforth referred to as R, (R Core Team 2022)) was kept in the analysis, upon inspection.

#### 3.1.2. Basic Statistics

Data Summary Statistics: Table 1 shows the summary statistics for the 37 AIAS Items. The AIAS items have intended rough classifications of C—creativity, MI—metacognition, P—practical, W—wisdom; however, as this is a new scale, the actual classifications will manifest themselves after the analysis of the data. Moreover, we expected items to overlap among these categories, as the categories themselves are related. The items that have been inverse-scored and therefore have been inverted are denoted by an “i” preceding the classification indicator in the item label.

#### 3.1.3. Intercorrelations

**Figure 1:**
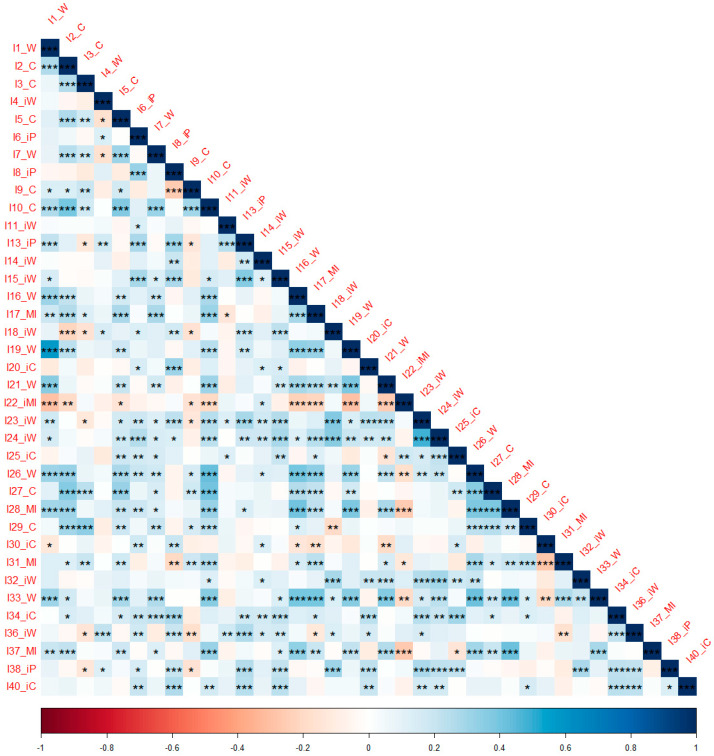
Correlation matrix plot and statistically significant correlations of the 37 AIAS items. Note—significance levels: 0 < *** < 0.001 < ** < 0.01 < * < 0.05. The darker the square, the stronger the relationship between variables.

As the AIAS items were measured on a Likert scale (1–5), given their ordinal nature and the fact that the data were not multi-normal (Mardia’s multivariate kurtosis = 1564.58, *p* < 0.001), Kendall rank correlations (Kendall’s tau) were used in the factor analysis. Inspection of the intercorrelation matrix of the 37 AIAS items given in Table 2 and its visualization together with the significance levels given in Figure 1 showed a number of statistically significant correlations above 0.3.

#### 3.1.4. Factor Analysis

To inspect the factor structure of the AIAS items, and to refine the AIAS if needed, exploratory factor analysis (EFA) was conducted. Through factor analysis, the aim was to explore the structures behind the intercorrelations between the AIAS items.

The overall KMO index of the 37 AIAS items was 0.8, with individual KMO indices greater than 0.60 for all the items except Item 11 (Item 11 KMO = 0.55), indicating that Item 11 was not suitable to be included in EFA (Kaiser–Meyer–Olkin measure of sampling adequacy, psych package (Revelle 2022), R.) After removing Item 11, the overall KMO index was 0.81, with individual KMO indices greater than 0.60, indicating the suitability of the correlation matrix of the remaining 36 AIAS items for EFA.

**Figure 2:**
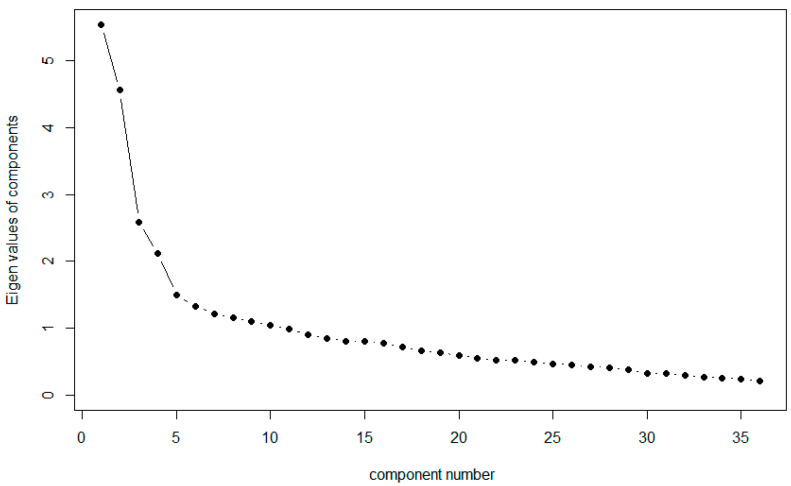
Scree plot of the intercorrelation matrix of the 36 AIAS items.

Choice of Factor Extraction and Rotation Methods and the Number of Factors to be Extracted: The Iterated Principal Axis (ipa) factor extraction method was chosen, given its suitability for analysis of non-normal data (Fabrigar et al. 1999; Costello and Osborne 2005), and squared multiple correlation (SMC) initialization was used. As the AIAS items were expected to have correlated factors, the oblimin oblique rotation method was chosen (Reise et al. 2000; Tabachnick et al. 2007, p. 646).) Inspection of the scree plot of the correlation matrix of the 36 AIAS items given in Figure 2 indicated that the optimal number of factors to be extracted was four. This was further confirmed by Velicer’s Minimum Average Partial Criteria (MAP) and Parallel Analysis results (implemented in the psych package (Revelle 2022), R) (Horn 1965; Tabachnick et al. 2007, pp. 644–45; Velicer et al. 2000).

**Figure 3:**
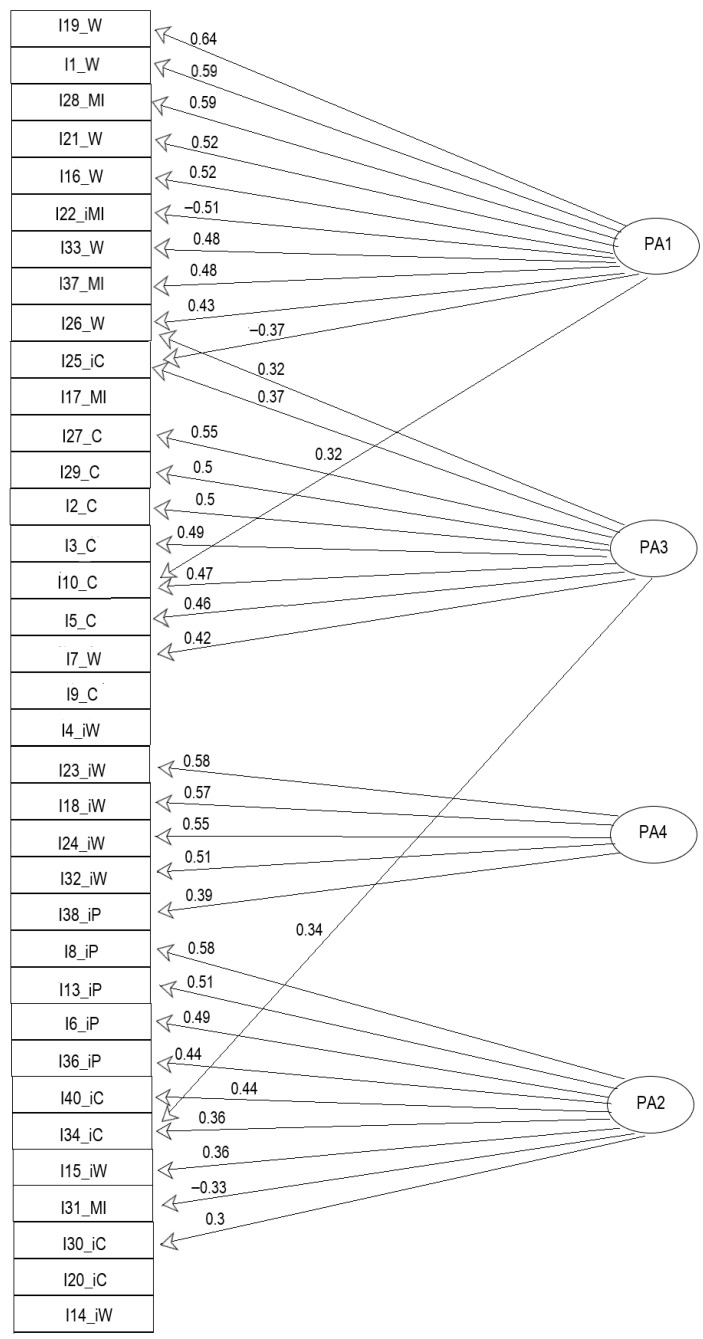
Path diagram of the salient loadings on the extracted factors of the factor model with 36 AIAS items.

Model Computation: The salient loading threshold was set to 0.3. The resulting factor model pattern matrix loadings are given in Table 3, with salient loadings in bold. The corresponding path diagram of the salient loadings on the extracted factors is presented in Figure 3.

Inspection of the pattern matrix loadings in Table 3 and the path diagram in Figure 3 showed that Items 4, 9, 14, 17, and 20 did not load saliently (threshold >= 0.3) on any of the four factors, and Items 25, 26, and 34 loaded saliently on two factors with high cross-loading ratios (>75%).

As a result, in the factor analysis, the AIAS items and the resulting factor models were systematically refined by successively removing, one at a time, the AIAS items that were either not loading on any of the factors or loading saliently on multiple factors with high cross-loading ratios. In each iteration, (i), KMO index values and factor loadings were inspected to decide which AIAS item should be removed; (ii) KMO index values of the remaining items were inspected to verify the suitability of the intercorrelation matrix of the remaining AIAS items for EFA; and (iii) the scree plot, as well as the MAP and the Parallel Analysis results, of the intercorrelation matrix of the remaining AIAS items were inspected to determine the number of factors to be extracted in the next factor model in the sequel. In each iteration, the methods in (iii) pointed to four as the optimal number of factors for the next factor model. As a result of this systematic iterative process, AIAS Items 4, 25, 14, 9, 20, 34, 17, and 26 were removed from the AIAS in the given order, 4 being the first and 26 being the last, until a finalized factor structure was obtained. The resulting AIAS had 28 items.

**Figure 4:**
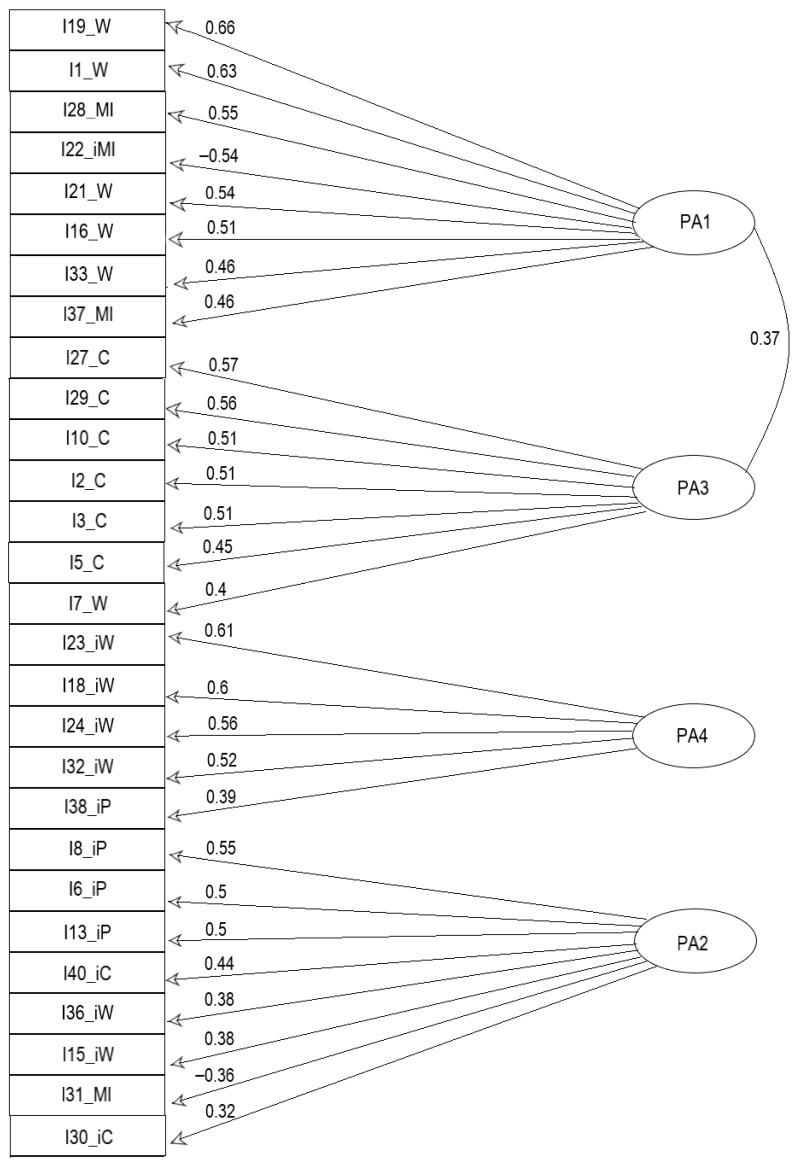
Path diagram of the salient loadings on the extracted factors of the factor model with 28 AIAS items.

Table 4 gives the pattern matrix loadings of the resulting factor model for the 28 AIAS items, with salient loadings in bold. Figure 4 gives the corresponding path diagram of the salient loadings on the extracted factors. Table 5 gives the proportion of, and cumulative total variance explained by, the extracted factors, as well as the correlations between the extracted factors.

Model Assessment: The resulting factor model had a simple structure (mean item complexity = 1.5), in which each AIAS item loaded saliently on one factor, and had the following factors:

PA1: Consisting of I19_W, I1_W, I28_MI, I22_iMI, I21_W, I16_W, I33_W, I37_MI
PA2: Consisting of I8_iP, I6_iP, I13_iP, I40_iC, I36_iW, I15_iW, I31_MI, I30_iC
PA3: Consisting of I27_C, I29_C, I10_C, I2_C, I3_C, I5_C, I7_W
PA4: Consisting of I23_iW, I18_iW, I24_iW, I32_iW, I38_iP.

All eight salient loadings on factor PA1, six out of seven salient loadings on factor PA3, four out of five salient loadings on factor PA4, and three out of eight salient loadings on factor PA2 were above the practical significance threshold value (0.45) for samples of size 150–200 (Hair et al. 2010, p. 177).

The overall model root mean square of residuals (RMSR) was 0.04, below the 0.08 cutoff for possible under-extraction, and 16% and 1% of the residual coefficients, in absolute value, respectively, exceeded values of 0.05 and 0.1, indicating a good model fit. Examination of the factor correlation matrix given in Table 5 showed that factors PA1 and PA3 correlated above the threshold (=0.3) of Tabachnick et al. (2007, pp. 644–45), and confirmed our initial thought regarding the need for the use of an oblique rotation method. The maximum factor correlation was 0.37, and this was the only factor correlation above 0.3. Factor PA1 accounted for 10% of the total variance and had an eigenvalue of 2.85. Factor PA3 accounted for 8% of the total variance and had an eigenvalue of 2.22. Factor PA4 accounted for 7% of the total variance and had an eigenvalue of 2.09. Factor PA2 accounted for 6% of total variance and had an eigenvalue of 1.8. All four factors cumulatively accounted for 32% of total variance. Given that EFA models account for a lower percentage of variance than corresponding PC models, and being aware that there are likely other yet-to-be discovered aspects of attitudinal intelligence and measures that capture their manifestations, we considered 32% of the variance being accounted for as an acceptable level.

The resulting four-factor model structure was robust to different factor extraction methods (ipa, minimum residual (minres), weighted least squares (wls), and generalized weighted least squares (gls)), combined with oblique rotation methods (Promax, oblimin).

We have interpreted these factors in the following way:

I. Wisdom (+): consideration of others’ and larger interests in addition to one’s own in life problem solving;
II. Adaptive intelligence (−): narcissistic self-preoccupation—self-centeredness and consideration only of oneself and one’s own interests in life problem solving;
III. Creativity (+): positive creativity in life problem solving;
IV. Wisdom (−): intellectual shallowness, self-aggrandizement, and narrowness in life problem solving.

Note that two of the factors stem from items that were positively scored—I and III—and two of the factors from items that were negatively scored—II and IV. The first and third factors represent attitudes that lead toward adaptively intelligent behavior, while the second and fourth factors represent attitudes that lead to behavior that is adaptively unintelligent.

### 3.2. Basic Statistics

Table 6 shows basic statistics for the study, using the now revised version of the AIAS.

As can be seen, the participants were a typical age for college populations, averaging 20.14 years of age. Their average GPA, 3.64 (out of 4.33—representing an A+), also is typical of academic averages in an era of grade inflation. With respect to the cognitive abilities measured by conventional tests, the mean reported scores are also fairly typical of highly selective institutions, with mean self-reported SAT scores of 717 (Reading) and 744 (Math) and a mean self-reported ACT score close to 33. All individual scores for tests were checked to ensure that they fell within the proper range for the variable being assessed.

### 3.3. Internal Consistency Reliabilities

Table 7 shows the internal consistency reliabilities. The coefficient alpha (internal consistency reliability for all possible split halves) of our new measure, the AIAS, was 0.76. The reliabilities of most of the other measures were in the 0.70 s. The Authoritarianism and Social Desirability scales had reliabilities in the 0.60 s, and the Critical Thinking scale and Creativity scale had reliabilities in the 0.80 s. The Dogmatism scale has a reliability of 0.91. Generally, reliabilities above 0.80 are considered good and reliabilities between 0.70 and 0.79 are considered acceptable (Cortina 1993). Reliabilities of less than 0.70 are considered less than desirable.

### 3.4. Intercorrelations

Table 8 shows intercorrelations among the various measures. There were a number of significant correlations of our new AIAS measure with other measures. The AIAS showed significant correlations in the 0.10 s with Letter Sets, Authoritarian Aggression (negative), Adventurousness, Liberalism, and Impression Management. Significant correlations were obtained in the 0.20 s with Conservatism (negative), Traditionalism (negative), Reflective Skepticism, Creativity, Artistic Interests, Emotionality, Social Desirability, and Self-Deceptive Enhancement. Significant correlations in the 0.30 s were obtained with Authoritarianism (negative), the Reflective element of Wisdom, Openness to Experience, and Intellect (as measured by typical performance). Significant correlations in the 0.40 s were obtained with Critical Thinking, Critical Openness, the Cognitive and Affective elements of Wisdom, Need for Cognition, and Dogmatism (negative). A significant correlation in the 0.50 s was obtained with Wisdom (overall score). Significant correlations were not obtained with GPA, ACT, SAT—Reading or Math, combined SAT and ACT, Figure Classification, or Imagination. Note that the correlational patterns were generally as expected (as described above).

### 3.5. Principal Component Analyses

Table 9, Table 10 and Table 11 show principal component analyses for the various assessments in this study.^1^ We have separated the analyses into three distinct ones for two reasons. First, this separation kept the ratios of participants to variables larger, as is desirable for such analyses. But also, second, we did so because the SAT and ACT were optional at the university in which the study was done at the time the study was done, resulting in a loss of participants for these variables. We lost roughly 15% of our participants due to listwise deletion when these standardized test variables were included in the principal component analyses. Thus, it was preferable to have separate analyses so that we would have principal component analyses that could include the entire sample for the other measures.

Table 9 shows the rotated principal components matrix for the Adaptive Intelligence Attitudes Scale (AIAS); psychometric assessments; Very Short Authoritarianism (VSA); Critical Thinking Dispositions Scale (CTDS); Wisdom (3D-WS-12); Creativity; Need for Cognition (NFC); Openness to Experience (OE); Dogmatism (DOG); and Balanced Inventory of Desirable Responding—that is, social desirability (BIDR).

Although factor loadings of 0.3 and above are often considered “significant”, we used a more stringent criterion of 0.5 to avoid false alarms (Type I error) (Hair et al. 2010, p. 177). Using 0.5 as a minimum cutoff for “significant” loadings on a component, the first component comprises the AIAS and five other tests: Critical Thinking, Wisdom, Creativity, Need for Cognition, and Openness to Experience. This component might be labeled (I) Intelligent Attitudes/Dispositions. The second component shows significant positive loadings for Letter Sets and Figure Classification and near but below significance level negative loading for Dogmatism. This component might be labeled (II) Fluid Intellectual Ability. The third component shows significant positive loadings for Authoritarianism and Social Desirability and significant negative loading for Openness to Experience and might be labeled (III) Deference to Authority.

Table 10 shows the rotated principal components matrix for the Adaptive Intelligence Attitudes Scale (AIAS); cumulative GPA, ACT and SAT to ACT (AC_SAT) conversion; Very Short Authoritarianism (VSA); Critical Thinking Dispositions Scale (CTDS); Wisdom (3D-WS-12); Creativity; Need for Cognition (NFC); Openness to Experience (OE); Dogmatism (DOG), and Balanced Inventory of Desirable Responding (BIDR). Again using 0.5 as the minimum cutoff for a “significant” loading, the first component contains the AIAS as well as Critical Thinking, Wisdom, Creativity, Need for Cognition, and Openness to Experience (as in Table 9) and shows near but below significance level positive loading for Social Desirability. This component again might be labeled (I) Intelligent Attitudes/Dispositions. The second component contains Authoritarianism, Dogmatism, and Social Desirability, and again might be labeled (II) Deference to Authority (the third component in the previous analysis). The third component contains GPA (grade point average) and SAT/ACT. This component might be labeled (III) Academic Ability/Achievement.

Table 11 shows rotated principal components for the two sets of the Ability/Achievement measure in the same analysis, plus the other measures. In particular, it shows the analysis for the Adaptive Intelligence Attitudes Scale (AIAS); psychometric assessments; cumulative GPA, ACT and SAT to ACT conversion (SAT_ACT); Very Short Authoritarianism (VSA); Critical Thinking Dispositions Scale (CTDS); Wisdom (3D-WS-12); Creativity; Need for Cognition (NFC); Openness to Experience (OE); Dogmatism (DOG); and Balanced Inventory of Desirable Responding (BIDR).

Once again using the 0.5 cutoff, Component 1 again contains the AIAS, plus Critical Thinking, Wisdom, Creativity, Need for Cognition, and Openness to Experience, and shows near but below significance level positive loading for Social Desirability. It is labeled (I) Intelligent Attitudes/Dispositions. Component 2 contains Letter Sets and Figure Classification and is labeled (II) Fluid Intellectual Abilities. Component 3 contains Authoritarianism and Social Desirability, and shows a near but below significance-level positive loading for Dogmatism and Openness to Experience, and is labeled (III) Deference to Authority. Component 4 contains GPA and SAT/ACT and is labeled Academic Ability/Achievement.

On the whole, the results of the three principal component analyses were quite consistent in yielding the four components shown in the last analysis:

(I) Intelligent Attitudes/Dispositions.
(II) Fluid Intellectual Abilities.
(III) Deference to Authority.
(IV) Academic Ability/Achievement.

## 4. Discussion

This study suggests that attitudes relevant to the deployment of adaptive intelligence are related to constructs that are also relevant to real-world adaptation, such as the need for cognition, wisdom, or openness to experience, but that they are distinct from fluid intelligence or scholastic aptitude. They represent views on how to deploy intelligence, not levels of intelligence. Someone could have a high level of intelligence but nevertheless choose to deploy it poorly or hardly at all; someone could have a lower level of intelligence but deploy what they have well.

Intelligence as an attitude has been a neglected construct—one that perhaps has not even been defined as a valid part of the construct, which has been viewed solely as an ability. Yet, in the end, what can be observed and measured in some way in the world is always intelligence as filtered through an individual’s attitude about its deployment, whether on a test, in school, or in solving problems in everyday life (Sternberg 2022). We, therefore, believe that intelligent attitudes need further study. Particular questions that might be asked are (a) how intelligent attitudes either facilitate or impede the display of intelligence as an ability, including on intelligence tests, (b) how students and others as well can be educated to deploy their intelligence more effectively for succeeding in the adaptive tasks they confront, (c) how students and others can be educated to deploy their intelligence more positively to make the world better, and (d) how intelligent attitudes relate to other constructs, such as of abilities and personality.

One might argue that it is hard to compare timed tests of intelligence as an ability with untimed tests of intelligence as an attitude or with personality, dispositions, wisdom, or other attributes that do not generate timed tests with so-called “objectively correct answers”. The mode of testing (power-based or speed-based), in this view, is confounded with the two kinds of constructs (intelligence or everything else). We would argue, however, that this is not a confounding. Many of the theorists of intelligence who have analyzed or produced intelligence tests (see, e.g., Cattell 1971; Carroll 1993; Ekstrom et al. 1976) have chosen, in our view, mistakenly (see, e.g., Sternberg and Grigorenko 2003), to view speed as intrinsic to fluid aspects of intelligence. They also have worked with a construct in which answers can be classified as right or wrong (although only approximately, as inductive reasoning problems do not have truly unique deductively correct answers). Not all intelligence test constructors have taken this point of view (e.g., Raven et al. 1992). But in using fluid ability tests derived from theories of intelligence in which mental speed is intrinsic to the definition of intelligence (e.g., Ekstrom et al. 1976), the tests must be timed, just as in using tests in which mental speed is not intrinsic to the definitions of the constructs, the tests must be power tests.

In the current view, attitudes always affect test scores. For example, the experience of taking an intelligence-type test or one of its proxies will be different if a high score can be used to earn admission to a prestigious university (especially in a country such as China, where the Gaokao is of utmost importance), versus a situation in which a high score can result in one’s being declared mentally competent (at least, in the United States) and therefore eligible for execution for a capital crime. When the senior author was younger, some 18-year-olds faked low scores on mental tests to avoid being drafted to fight (in Vietnam). The examples do not have to be as extreme. For some people, their scores on standardized tests may determine their future (say, future professional scholars), whereas for other people, the scores will matter little (say, for future professional gardeners). This is not to say that abilities *are* attitudes, but rather, that scores on ability tests are not pure—they can be influenced by attitudes.

It might be suggested that tests of attitudes somehow could be scored in a way to create objectively better and worse attitudes about specific issues in the world, as might be the case for, say, a test of emotional intelligence. But such a suggestion, which the authors have actually received, is bound to fail. Try telling people that there are “correct” or even “better” and “worse” attitudes about any important issue—abortion, capital punishment, political ideology—and you likely will not get very far.

The patterns of correlations and rotated principal components (as well as rotated factors from principal axis factor analyses with oblimin rotation) largely corresponded to our expectations. In general, we found separate factors for intelligent attitudes/dispositions, fluid intellectual abilities, academic ability/achievement, and deference to authority. However, we found that intelligent attitudes correlate positively, to some extent, with one aspect of deference to authority, social desirability, perhaps because intelligent attitudes are intrinsically socially desirable, at least in the US society in which the measurements took place.

The study we conducted had some limitations that must be noted.

First, the sample was from a highly selective university, at least by the standards of the United States. It is the sample that was available to us in the absence of extramural funding of participants and in the presence of a subject pool in our university. The mean SAT score (sum of Reading and Math) was 1105 in 2023 (https://wisevoter.com/state-rankings/sat-scores-by-state/#average-sat-scores, accessed on 2 April 2024; our mean was 1461. Results might have been different in an intellectually more diverse sample. That said, our concern was not with the aspect of intelligence as an ability measured by the SAT, but rather with (adaptive) intelligence as attitude, and it is not clear how different our sample would have been in this respect from one with different SAT scores.

Second, we present this work only as a single study with just short of 200 (*N* = 187) participants. In future work, more participants would need to be tested, ideally, in a variety of cultures. Moreover, our participants were college students, generally in the age range of 18–22, and further work would be needed to extend the age range to see whether results are the same across age groups.

Third, however modifiable abilities may or may not be, attitudes certainly are at least somewhat modifiable. So it may be that, with intervention, perhaps even fairly rudimentary intervention, attitudes could be changed and made more favorable for positive deployment of adaptive intelligence. That remains to be seen.

Fourth, our measure was a typical-performance Likert-scale assessment. There is always a chance that at least some participants answered in ways they thought the experimenters would want to hear. We examined the distributions of scores on the Social Desirability and Self-Deceptive Enhancement subscales to investigate this possibility. We found normal distributions for both and no high-end single or multiple outliers that would suggest people were faking. Indeed, the data were collected anonymously and for course credit simply by virtue of participation, so there was no obvious incentive to fake.

We believe that the notion of adaptively intelligent attitudes is an important one in today’s world and has important implications for understanding intelligence as it applies in everyday life. It is important because many people, even intelligent ones, fail sufficiently to deploy their intelligence, and some who do deploy it may deploy it in ways that are not conducive to adaptation. For example, political leaders whose only goal is to stay in power, or who start wars to achieve internal cohesion, are not deploying their intelligence in an optimal way. We plan to investigate further light and dark deployments of intelligence, and hope that in conjunction with this work, we can better understand how to encourage people to deploy their intelligence in the interest of making the world a better place.

We emphasize that our goal is *not* to suggest that intelligence is merely a set of attitudes rather than a set of abilities. We do not interpret either our theoretical proposals or empirical data as suggesting that any particular theory of intelligence, or theories of intelligence, in general, are in need of correction. We simply did not address the issue of the adequacy of any particular psychometric, cognitive, biological, cultural, or other theory (see reviews of theories in Sternberg 2020). Rather, we have suggested that when intelligence is viewed, as it historically has been, in terms of adaptation to the environment (Binet and Simon 1916; Gottfredson 1997; “Intelligence and Its Measurement” 1921; Spearman 1923; Wechsler 1940), attitudes matter as well as abilities because attitudes are largely, although not exclusively, what determine how intelligence is deployed. Understanding intelligent attitudes can help close the conceptual as well as predictive gaps between intelligence as conceptualized, measured, and successfully deployed in the world. We, therefore, suggest that intelligent attitudes be explored seriously as a way of better understanding how intelligence functions in the everyday world, not just in controlled and sometimes contrived situations.

This work was described at the beginning of the article as a preliminary to a construct validation rather than as itself a construct validation of the notion of intelligence as having an attitudinal component. What might future research look like that would begin a more advanced process of construct validation? It would seek to establish a nomological network for intelligence as an attitude (Cronbach and Meehl 1955) and indicate whether the conceptual differences among constructs are matched by statistical differences.

First, one could examine a broader range of intellectual abilities. The abilities are cognitive, and other sets of developed skills, as opposed to attitudes toward the use of those skills, could be explored. A first step would be to compare intelligence as an attitude to more of the cognitive abilities that comprise intelligence, at minimum, crystallized, as well as the fluid ability that was measured here (see Cattell 1971) and other abilities described in Carroll’s (1993) conceptualization of the Cattell–Horn–Carroll (McGrew 2009) conception. These other abilities might include various speed, perceptual, and memory factors that were not considered here.

Second, one could examine rational thinking. One has a certain level of ability to think rationally, but attitudinally, one may or may not choose to use this ability. Often, when problems are high-stakes or are emotionally laden, people do not think as rationally as they are capable of thinking because they are too swayed by answers they want rather than answers that would be, from a rational point of view, best. Rational thinking, as an ability, can yield tests that have more and less rational answers, as objectively defined, and as have been assessed by Stanovich and colleagues. A second step, therefore, could be to compare intelligence as an attitude to aspects of rational thinking, as studied by Stanovich and his colleagues (Stanovich 2009, 2021; Stanovich and West 2000; Stanovich et al. 2013).

Third, one could examine personality traits. Personality traits are fairly stable characteristics of how people react to situations, in general, whereas attitudes are more malleable and are thoughts relevant to how people will react in particular situations. A third step, therefore, could be to compare intelligence as an attitude to personality as conceptualized in the five-factor theory—the so-called “OCEAN” theory specifying as factors openness, conscientiousness, extraversion, agreeableness, and neuroticism (McCrae and Costa 2008; Poropat 2009).

Fourth, one could examine motivation. Motivation is the desire or willingness of someone to do something or a set of things. Attitudes may lead to motivations, as when an individual who has conservative (or liberal) attitudes is motivated to vote for a political candidate with similar attitudes. A fourth step, therefore, could be to compare intelligence as an attitude to aspects of motivation, for example, in Ryan and Deci’s (2017) self-determination theory of motivation. One would then be examining how intelligence as an attitude compares to needs for autonomy, competence, and relatedness.

Fifth, one could look at future behavior. A fifth and perhaps most important step could be to relate intelligence as an attitude to future performances in the world—in school, on the job, or in one’s personal life (such as health maintenance, marital success, or longevity).

Intelligence tests represent one deployment of intelligence. But for the emotionally charged, high-stakes, and sometimes even life-threatening situations people confront in their lives, intelligent attitudes can make the difference between success and failure in adaptation. We therefore recommend that they be considered, as well as abilities, in our understanding of how to conceptualize, measure, and teach for intelligence.

## Figures and Tables

**Table 1:** Summary statistics for the 37 AIAS items.

	n	Mean	sd	Median	Min	Max	Skew	Kurtosis
I1_W	187	4.06	0.81	4	2	5	−0.53	−0.33
I2_C	187	3.63	0.79	4	1	5	−0.02	−0.16
I3_C	187	3.13	0.97	3	1	5	0.16	−0.50
I4_iW	187	3.28	1.01	3	1	5	−0.14	−0.56
I5_C	187	3.68	0.76	4	2	5	−0.14	−0.34
I6_iP	187	3.34	1.06	3	1	5	−0.22	−0.55
I7_W	187	3.70	0.83	4	2	5	−0.40	−0.32
I8_iP	187	3.01	1.08	3	1	5	−0.05	−0.70
I9_C	187	3.24	1.01	3	1	5	−0.17	−0.54
I10_C	187	3.76	0.90	4	1	5	−0.55	−0.03
I11_iW	187	2.66	1.03	3	1	5	0.11	−0.73
I13_iP	187	3.61	1.11	4	1	5	−0.40	−0.82
I14_iW	187	3.25	1.09	3	1	5	−0.16	−0.90
I15_iW	187	3.59	1.08	4	1	5	−0.60	−0.24
I16_W	187	3.90	0.87	4	2	5	−0.49	−0.43
I17_MI	187	3.84	0.81	4	1	5	−0.43	0.09
I18_iW	187	3.75	0.95	4	1	5	−0.43	−0.41
I19_W	187	3.99	0.84	4	1	5	−0.59	0.08
I20_iC	187	3.23	0.90	3	1	5	−0.03	−0.12
I21_W	187	3.98	0.85	4	2	5	−0.54	−0.33
I22_iMI	187	2.54	0.96	2	1	5	0.47	−0.12
I23_iW	187	3.66	1.12	4	1	5	−0.45	−0.63
I24_iW	187	3.81	1.04	4	1	5	−0.55	−0.52
I25_iC	187	2.93	1.00	3	1	5	0.01	−0.62
I26_W	187	3.83	0.86	4	1	5	−0.47	−0.13
I27_C	187	3.52	0.99	4	1	5	−0.42	−0.32
I28_MI	187	3.67	0.81	4	1	5	−0.40	0.00
I29_C	187	3.02	0.96	3	1	5	0.21	−0.67
I30_iC	187	2.31	0.99	2	1	5	0.33	−0.41
I31_MI	187	3.44	0.97	3	1	5	−0.12	−0.62
I32_iW	187	3.86	0.95	4	1	5	−0.67	0.06
I33_W	187	3.86	0.79	4	1	5	−0.58	0.44
I34_iC	187	3.32	0.95	3	1	5	−0.25	−0.47
I36_iW	187	3.02	1.08	3	1	5	0.09	−0.73
I37_MI	187	3.64	0.87	4	1	5	−0.46	0.20
I38_iP	187	3.43	1.01	4	1	5	−0.27	−0.58
I40_iC	187	3.18	1.01	3	1	5	−0.14	−0.57

**Table 2:** Intercorrelation matrix of the 37 AIAS items.

	I1_W	I2_C	I3_C	I4_iW	I5_C	I6_iP	I7_W	I8_iP	I9_C	I10_C	I11_iW	I13_iP	I14_iW	I15_iW	I16_W	I17_MI	I18_iW	I19_W	I20_iC	I21_W	I22_iMI	I23_iW	I24_iW	I25_iC	I26_W	I27_C	I28_MI	I29_C	I30_iC	I31_MI	I32_iW	I33_W	I34_iC	I36_iW	I37_MI	I38_iP	I40_iC
I1_W	1.00	0.26	0.07	0.04	0.11	0.09	0.08	−0.05	0.13	0.26	0.02	0.22	0.02	0.15	0.30	0.19	0.09	0.58	−0.11	0.36	−0.29	0.18	0.15	−0.05	0.30	0.10	0.29	0.05	−0.13	0.12	0.12	0.31	0.01	0.05	0.19	0.09	0.06
I2_C	0.26	1.00	0.25	−0.05	0.26	0.05	0.25	−0.06	0.14	0.39	0.01	0.11	−0.01	−0.02	0.22	0.26	−0.22	0.23	0.04	0.06	−0.19	0.00	0.05	0.03	0.33	0.36	0.31	0.29	0.00	0.13	−0.04	0.16	0.15	−0.03	0.22	−0.04	0.07
I3_C	0.07	0.25	1.00	−0.08	0.17	−0.04	0.18	−0.12	0.19	0.16	0.02	−0.12	−0.01	−0.03	0.07	0.13	−0.16	0.06	−0.09	−0.02	0.03	−0.12	−0.01	0.08	0.08	0.23	0.06	0.35	0.07	0.20	−0.02	0.03	0.11	−0.14	0.02	−0.13	0.01
I4_iW	0.04	−0.05	−0.08	1.00	−0.16	0.13	−0.16	0.11	−0.10	0.00	0.05	0.16	0.09	0.01	0.03	0.07	0.15	0.09	0.00	0.02	−0.08	−0.02	0.04	−0.11	0.05	0.01	0.11	−0.10	−0.05	0.00	0.09	0.07	0.04	0.22	0.02	0.13	0.06
I5_C	0.11	0.26	0.17	−0.16	1.00	0.00	0.30	−0.10	0.16	0.38	−0.05	−0.04	−0.08	0.10	0.17	0.28	0.09	0.18	−0.03	0.21	−0.15	0.16	0.17	0.18	0.28	0.33	0.20	0.18	0.01	0.17	0.10	0.29	0.16	−0.03	0.18	0.04	−0.03
I6_iP	0.09	0.05	−0.04	0.13	0.00	1.00	−0.03	0.33	−0.11	0.12	0.13	0.27	0.11	0.28	0.11	0.03	0.14	0.07	0.14	−0.02	−0.06	0.18	0.25	0.16	0.19	0.07	0.19	0.06	0.17	−0.09	0.04	0.10	0.19	0.18	−0.01	0.15	0.20
I7_W	0.08	0.25	0.18	−0.16	0.30	−0.03	1.00	0.06	0.06	0.32	0.02	−0.02	0.04	0.13	0.18	0.28	0.11	0.11	0.04	0.17	−0.04	0.13	0.15	0.16	0.17	0.15	0.13	0.21	0.02	0.03	0.08	0.26	0.21	−0.11	0.13	0.00	0.07
I8_iP	−0.05	−0.06	−0.12	0.11	−0.10	0.33	0.06	1.00	−0.26	0.01	0.08	0.29	0.19	0.27	−0.04	0.00	0.16	−0.03	0.30	−0.08	0.03	0.22	0.14	0.10	−0.05	−0.05	0.03	−0.06	0.20	−0.20	0.05	−0.01	0.28	0.21	−0.04	0.21	0.27
I9_C	0.13	0.14	0.19	−0.10	0.16	−0.11	0.06	−0.26	1.00	0.29	−0.04	−0.13	−0.10	−0.11	0.12	0.07	−0.16	0.10	−0.10	0.01	−0.15	−0.13	−0.08	−0.01	0.16	0.17	0.07	0.15	−0.08	0.18	0.03	0.00	−0.10	−0.17	0.11	−0.16	0.00
I10_C	0.26	0.39	0.16	0.00	0.38	0.12	0.32	0.01	0.29	1.00	0.00	0.11	0.11	0.16	0.28	0.31	0.04	0.25	0.12	0.31	−0.21	0.25	0.23	0.07	0.43	0.41	0.33	0.22	−0.07	0.25	0.13	0.35	0.12	0.05	0.32	0.01	0.16
I11_iW	0.02	0.01	0.02	0.05	−0.05	0.13	0.02	0.08	−0.04	0.00	1.00	0.24	−0.10	0.12	0.00	−0.14	−0.05	−0.04	−0.04	−0.09	0.07	0.12	0.00	0.12	−0.05	0.04	0.04	0.07	0.07	−0.05	−0.02	−0.08	0.09	0.18	−0.05	0.06	0.10
I13_iP	0.22	0.11	−0.12	0.16	−0.04	0.27	−0.02	0.29	−0.13	0.11	0.24	1.00	0.18	0.37	0.08	−0.01	0.21	0.17	0.09	0.11	0.03	0.28	0.18	0.01	0.09	0.05	0.13	−0.04	−0.04	−0.08	0.05	0.06	0.17	0.26	0.04	0.25	0.25
I14_iW	0.02	−0.01	−0.01	0.09	−0.08	0.11	0.04	0.19	−0.10	0.11	−0.10	0.18	1.00	0.12	−0.04	−0.08	0.12	0.06	0.13	0.02	0.15	0.18	0.19	0.09	0.13	0.07	0.07	−0.02	0.13	−0.01	0.14	0.03	0.17	0.15	−0.02	0.11	0.08
I15_iW	0.15	−0.02	−0.03	0.01	0.10	0.28	0.13	0.27	−0.11	0.16	0.12	0.37	0.12	1.00	0.05	0.07	0.22	0.11	0.15	0.16	−0.07	0.25	0.28	0.19	0.12	−0.01	0.08	−0.01	0.02	−0.06	0.10	0.13	0.21	0.17	0.13	0.21	0.23
I16_W	0.30	0.22	0.07	0.03	0.17	0.11	0.18	−0.04	0.12	0.28	0.00	0.08	−0.04	0.05	1.00	0.28	0.02	0.34	−0.07	0.29	−0.23	0.06	0.13	−0.04	0.40	0.26	0.40	0.13	−0.13	0.16	0.02	0.40	0.12	0.04	0.33	−0.04	0.10
I17_MI	0.19	0.26	0.13	0.07	0.28	0.03	0.28	0.00	0.07	0.31	−0.14	−0.01	−0.08	0.07	0.28	1.00	0.04	0.33	−0.01	0.24	−0.21	0.12	0.24	0.13	0.34	0.21	0.22	0.10	−0.17	0.23	0.10	0.40	0.15	−0.12	0.19	−0.01	−0.04
I18_iW	0.09	−0.22	−0.16	0.15	0.09	0.14	0.11	0.16	−0.16	0.04	−0.05	0.21	0.12	0.22	0.02	0.04	1.00	0.05	0.09	0.19	0.04	0.38	0.35	0.06	0.09	−0.04	0.04	−0.20	−0.05	0.03	0.28	0.13	0.12	0.13	−0.02	0.31	0.05
I19_W	0.58	0.23	0.06	0.09	0.18	0.07	0.11	−0.03	0.10	0.25	−0.04	0.17	0.06	0.11	0.34	0.33	0.05	1.00	−0.05	0.38	−0.30	0.13	0.20	−0.11	0.31	0.16	0.39	0.08	−0.12	0.12	0.11	0.36	0.02	0.07	0.22	0.07	0.04
I20_iC	−0.11	0.04	−0.09	0.00	−0.03	0.14	0.04	0.30	−0.10	0.12	−0.04	0.09	0.13	0.15	−0.07	−0.01	0.09	−0.05	1.00	−0.03	0.06	0.23	0.17	0.02	0.01	−0.02	−0.08	0.00	0.02	−0.01	0.19	−0.05	0.24	0.15	−0.11	0.23	0.20
I21_W	0.36	0.06	−0.02	0.02	0.21	−0.02	0.17	−0.08	0.01	0.31	−0.09	0.11	0.02	0.16	0.29	0.24	0.19	0.38	−0.03	1.00	−0.26	0.22	0.17	−0.15	0.21	0.01	0.27	0.02	−0.18	0.14	0.21	0.36	0.00	0.01	0.25	0.01	0.03
I22_iMI	−0.29	−0.19	0.03	−0.08	−0.15	−0.06	−0.04	0.03	−0.15	−0.21	0.07	0.03	0.15	−0.07	−0.23	−0.21	0.04	−0.30	0.06	−0.26	1.00	0.03	−0.06	0.17	−0.17	−0.04	−0.26	0.05	0.11	−0.12	0.12	−0.19	0.05	0.11	−0.26	0.05	−0.06
I23_iW	0.18	0.00	−0.12	−0.02	0.16	0.18	0.13	0.22	−0.13	0.25	0.12	0.28	0.18	0.25	0.06	0.12	0.38	0.13	0.23	0.22	0.03	1.00	0.49	0.16	0.16	0.03	0.04	0.01	−0.04	0.09	0.25	0.15	0.25	0.15	−0.02	0.27	0.17
I24_iW	0.15	0.05	−0.01	0.04	0.17	0.25	0.15	0.14	−0.08	0.23	0.00	0.18	0.19	0.28	0.13	0.24	0.35	0.20	0.17	0.17	−0.06	0.49	1.00	0.22	0.21	0.07	0.06	0.02	−0.04	0.08	0.29	0.23	0.20	0.12	0.09	0.23	0.17
I25_iC	−0.05	0.03	0.08	−0.11	0.18	0.16	0.16	0.10	−0.01	0.07	0.12	0.01	0.09	0.19	−0.04	0.13	0.06	−0.11	0.02	−0.15	0.17	0.16	0.22	1.00	−0.01	0.17	−0.05	0.06	0.16	−0.12	0.18	0.04	0.29	0.09	−0.14	0.22	−0.01
I26_W	0.30	0.33	0.08	0.05	0.28	0.19	0.17	−0.05	0.16	0.43	−0.05	0.09	0.13	0.12	0.40	0.34	0.09	0.31	0.01	0.21	−0.17	0.16	0.21	−0.01	1.00	0.35	0.39	0.22	−0.08	0.28	0.20	0.41	0.11	0.00	0.30	0.02	0.08
I27_C	0.10	0.36	0.23	0.01	0.33	0.07	0.15	−0.05	0.17	0.41	0.04	0.05	0.07	−0.01	0.26	0.21	−0.04	0.16	−0.02	0.01	−0.04	0.03	0.07	0.17	0.35	1.00	0.38	0.25	0.08	0.16	0.00	0.18	0.14	−0.01	0.20	−0.02	0.09
I28_MI	0.29	0.31	0.06	0.11	0.20	0.19	0.13	0.03	0.07	0.33	0.04	0.13	0.07	0.08	0.40	0.22	0.04	0.39	−0.08	0.27	−0.26	0.04	0.06	−0.05	0.39	0.38	1.00	0.19	0.00	0.20	0.04	0.42	0.09	0.01	0.42	−0.04	0.01
I29_C	0.05	0.29	0.35	−0.10	0.18	0.06	0.21	−0.06	0.15	0.22	0.07	−0.04	−0.02	−0.01	0.13	0.10	−0.20	0.08	0.00	0.02	0.05	0.01	0.02	0.06	0.22	0.25	0.19	1.00	−0.03	0.23	0.03	0.14	0.13	0.02	0.07	−0.07	0.16
I30_iC	−0.13	0.00	0.07	−0.05	0.01	0.17	0.02	0.20	−0.08	−0.07	0.07	−0.04	0.13	0.02	−0.13	−0.17	−0.05	−0.12	0.02	−0.18	0.11	−0.04	−0.04	0.16	−0.08	0.08	0.00	−0.03	1.00	−0.27	−0.12	−0.18	0.10	0.04	−0.06	−0.02	0.10
I31_MI	0.12	0.13	0.20	0.00	0.17	−0.09	0.03	−0.20	0.18	0.25	−0.05	−0.08	−0.01	−0.06	0.16	0.23	0.03	0.12	−0.01	0.14	−0.12	0.09	0.08	−0.12	0.28	0.16	0.20	0.23	−0.27	1.00	0.11	0.26	−0.06	−0.17	0.07	−0.11	−0.07
I32_iW	0.12	−0.04	−0.02	0.09	0.10	0.04	0.08	0.05	0.03	0.13	−0.02	0.05	0.14	0.10	0.02	0.10	0.28	0.11	0.19	0.21	0.12	0.25	0.29	0.18	0.20	0.00	0.04	0.03	−0.12	0.11	1.00	0.20	0.12	0.10	0.08	0.28	0.01
I33_W	0.31	0.16	0.03	0.07	0.29	0.10	0.26	−0.01	0.00	0.35	−0.08	0.06	0.03	0.13	0.40	0.40	0.13	0.36	−0.05	0.36	−0.19	0.15	0.23	0.04	0.41	0.18	0.42	0.14	−0.18	0.26	0.20	1.00	0.07	−0.04	0.29	0.10	−0.03
I34_iC	0.01	0.15	0.11	0.04	0.16	0.19	0.21	0.28	−0.10	0.12	0.09	0.17	0.17	0.21	0.12	0.15	0.12	0.02	0.24	0.00	0.05	0.25	0.20	0.29	0.11	0.14	0.09	0.13	0.10	−0.06	0.12	0.07	1.00	0.22	0.01	0.22	0.23
I36_iW	0.05	−0.03	−0.14	0.22	−0.03	0.18	−0.11	0.21	−0.17	0.05	0.18	0.26	0.15	0.17	0.04	−0.12	0.13	0.07	0.15	0.01	0.11	0.15	0.12	0.09	0.00	−0.01	0.01	0.02	0.04	−0.17	0.10	−0.04	0.22	1.00	0.00	0.21	0.23
I37_MI	0.19	0.22	0.02	0.02	0.18	−0.01	0.13	−0.04	0.11	0.32	−0.05	0.04	−0.02	0.13	0.33	0.19	−0.02	0.22	−0.11	0.25	−0.26	−0.02	0.09	−0.14	0.30	0.20	0.42	0.07	−0.06	0.07	0.08	0.29	0.01	0.00	1.00	−0.09	0.01
I38_iP	0.09	−0.04	−0.13	0.13	0.04	0.15	0.00	0.21	−0.16	0.01	0.06	0.25	0.11	0.21	−0.04	−0.01	0.31	0.07	0.23	0.01	0.05	0.27	0.23	0.22	0.02	−0.02	−0.04	−0.07	−0.02	−0.11	0.28	0.10	0.22	0.21	−0.09	1.00	0.13
I40_iC	0.06	0.07	0.01	0.06	−0.03	0.20	0.07	0.27	0.00	0.16	0.10	0.25	0.08	0.23	0.10	−0.04	0.05	0.04	0.20	0.03	−0.06	0.17	0.17	−0.01	0.08	0.09	0.01	0.16	0.10	−0.07	0.01	−0.03	0.23	0.23	0.01	0.13	1.00

**Table 3:** Pattern matrix loadings of the factor model with 36 AIAS items.

	PA1	PA3	PA4	PA2
I19_W	**0.64**	−0.02	0.06	0.04
I1_W	**0.59**	−0.04	0.06	0.05
I28_MI	**0.59**	0.2	−0.14	0.14
I21_W	**0.52**	−0.11	0.27	−0.13
I16_W	**0.52**	0.16	−0.03	0.03
I22_iMI	**−0.51**	0.06	0.13	0.03
I33_W	**0.48**	0.14	0.28	−0.12
I37_MI	**0.48**	0.1	−0.09	0
I26_W	**0.43**	**0.32**	0.11	0.02
I25_iC	**−0.37**	**0.37**	0.29	0.1
I17_MI	0.29	0.27	0.22	−0.17
I27_C	0.14	**0.55**	−0.1	0.07
I29_C	−0.01	**0.5**	−0.1	−0.01
I2_C	0.25	**0.5**	−0.2	0.08
I3_C	−0.09	**0.49**	−0.12	−0.13
I10_C	**0.32**	**0.47**	0.14	0.03
I5_C	0.08	**0.46**	0.24	−0.18
I7_W	0	**0.42**	0.22	−0.08
I9_C	0.11	0.26	−0.13	−0.26
I4_iW	0.22	−0.23	−0.02	0.22
I23_iW	0.02	0.03	**0.58**	0.17
I18_iW	0.05	−0.21	**0.57**	0.07
I24_iW	0.07	0.1	**0.55**	0.12
I32_iW	0.01	0.02	**0.51**	−0.07
I38_iP	−0.06	−0.08	**0.39**	0.26
I8_iP	−0.07	−0.03	0.09	**0.58**
I13_iP	0.24	−0.13	0.1	**0.51**
I6_iP	0.1	0.07	0.04	**0.49**
I36_iW	0.04	−0.1	0.07	**0.44**
I40_iC	0.04	0.12	−0.02	**0.44**
I34_iC	−0.13	**0.34**	0.2	**0.36**
I15_iW	0.11	0.02	0.26	**0.36**
I31_MI	0.21	0.18	0.13	**−0.33**
I30_iC	−0.25	0.17	−0.18	**0.3**
I20_iC	−0.16	0.07	0.22	0.26
I14_iW	−0.04	0.04	0.15	0.26

Note: Salient loadings are in bold.

**Table 4:** Pattern matrix loadings of the factor model with 28 AIAS items.

	PA1	PA3	PA4	PA2
I19_W	**0.66**	−0.02	0.01	0.03
I1_W	**0.63**	−0.05	0.04	0.05
I28_MI	**0.55**	0.23	−0.13	0.11
I22_iMI	**−0.54**	0.07	0.17	0.01
I21_W	**0.54**	−0.08	0.24	−0.15
I16_W	**0.51**	0.15	−0.05	0.02
I33_W	**0.46**	0.16	0.24	−0.14
I37_MI	**0.46**	0.12	−0.09	0
I27_C	0.06	**0.57**	−0.06	0.07
I29_C	−0.1	**0.56**	−0.03	−0.01
I10_C	0.24	**0.51**	0.19	0.04
I2_C	0.2	**0.51**	−0.18	0.09
I3_C	−0.14	**0.51**	−0.08	−0.12
I5_C	0.06	**0.45**	0.24	−0.14
I7_W	−0.01	**0.4**	0.21	−0.05
I23_iW	−0.01	0.07	**0.61**	0.16
I18_iW	0.01	−0.16	**0.6**	0.05
I24_iW	0.04	0.12	**0.56**	0.13
I32_iW	−0.01	0.02	**0.52**	−0.11
I38_iP	−0.05	−0.09	**0.39**	0.23
I8_iP	−0.09	−0.02	0.11	**0.55**
I6_iP	0.06	0.07	0.06	**0.5**
I13_iP	0.19	−0.09	0.14	**0.5**
I40_iC	−0.03	0.16	0.05	**0.44**
I36_iW	0.01	−0.07	0.11	**0.38**
I15_iW	0.11	0.01	0.26	**0.38**
I31_MI	0.13	0.25	0.16	**−0.36**
I30_iC	−0.25	0.15	−0.16	**0.32**

Note: Salient loadings are in bold.

**Table 5:** Total variance explained and factor correlations of the extracted factors of the factor model with 28 AIAS items.

Total Variance Explained					Factor Correlations				
	PA1	PA3	PA4	PA2		PA1	PA3	PA4	PA2
Eigenvalues	2.85	2.22	2.09	1.8	PA1	1	0.37	0.25	0.03
Proportion	0.1	0.08	0.07	0.06	PA3	0.37	1	0.02	−0.01
Cumulative	0.1	0.18	0.26	0.32	PA4	0.25	0.02	1	0.2
					PA2	0.03	−0.01	0.2	1

**Table 6:** Mean scores and standard deviations.

	Mean	Standard Deviation	N
Age	20.14	1.29	187
GPA	3.64	0.35	183
Year of study	2.62	1.03	180
ACT	32.89	2.48	65
SAT—Reading	715.58	59.78	113
SAT—Math	743.45	72.81	113
SAT_ACT (scaled on ACT scale)	32.71	3.18	154
Letter Sets	9.97	2.91	187
Figure Classification	5.43	2.38	187
VSA	21.26	7.08	187
VSA—Dimension 1—Conservatism	7.35	2.78	187
VSA—Dimension 2—Traditionalism	4.87	4.00	187
VSA—Dimension 3—Authoritarian Aggression	9.03	2.86	187
Critical Thinking	46.02	5.43	184
Critical Thinking—Dimension 1—Critical Openness	29.34	3.40	185
Critical Thinking—Dimension 2—Reflective Skepticism	16.65	2.66	186
Wisdom	40.82	6.42	187
Wisdom—Dimension 1—Cognitive	13.13	2.93	187
Wisdom—Dimension 2—Reflective	13.13	2.89	187
Wisdom—Dimension 3—Affective	14.56	2.51	187
Creativity	28.09	5.52	184
NFC	59.68	9.04	183
OE	45.62	6.62	184
OE—Dimension 1—Imagination	8.04	1.85	187
OE—Dimension 2—Artistic Interests	8.64	1.68	186
OE—Dimension 3—Emotionality	7.84	2.01	186
OE—Dimension 4—Adventurousness	5.88	1.79	186
OE—Dimension 5—Intellect	7.50	2.20	187
OE—Dimension 6—Liberalism	7.78	1.88	187
DOG	67.72	23.79	183
AIAS	98.09	9.82	187
BIDR	57.86	9.53	184
BIDR—Dimension 1—SDE	30.37	5.43	187
BIDR—Dimension 2—IM	27.49	5.97	184

Note: SAT_ACT = ACT and SAT to ACT conversion; VSA= Very Short Authoritarianism (2018); NFC = Need for Cognition; OE = Openness to Experience; DOG = Dogmatism; AIAS = Adaptive Intelligence Attitudes Scale; BIDR = Balanced Inventory of Desirable Responding; SDE = Self-Deceptive Enhancement; IM = Impression Management.

**Table 7:** Internal consistency reliabilities (coefficient alpha).

Test	Coefficient Alpha Reliability	Number of Items
Letter Sets	0.77	15
Figure Classification	0.70	14
AIAS	0.76	28
VSA	0.65	6
CTDS	0.83	11
Wisdom	0.70	12
Creativity	0.80	8
NFC	0.76	18
OE	0.74	12
DOG	0.91	20
BIDR	0.69	16

Note: SAT_ACT = ACT and SAT to ACT conversion; VSA = Very Short Authoritarianism (2018); CTDS = Critical Thinking Dispositions Scale; NFC = Need for Cognition; OE = Openness to Experience; DOG = Dogmatism; AIAS = Adaptive Intelligence Attitudes Scale; BIDR = Balanced Inventory of Desirable Responding.

**Table 8:** Intercorrelations.

		**1**	**2**	**3**	**4**	**5**	**6**	**7**	**8**	**9**	**10**	**11**	**12**	**13**	**14**	**15**	**16**
1	AIAS	1															
2	GPA	0.07	1														
3	ACT	−0.07	0.30 *	1													
4	SATReading	0.16	0.40 **	0.39	1												
5	SATMath	0.09	0.40 **	0.50 *	0.57 **	1											
6	SAT_ACT	0.10	0.43 **	0.85 **	0.85 **	0.91 **	1										
7	LS	0.18 *	0.03	0.20	0.14	0.12	0.18 *	1									
8	FC	0.14	0.07	0.26 *	0.23 *	0.24 **	0.29 **	0.50 **	1								
9	VSA	−0.31 **	0.03	0.02	−0.09	0.02	−0.04	−0.16 *	−0.23 **	1							
10	Conservatism	−0.28 **	0.01	0.00	−0.07	0.03	−0.02	−0.09	−0.20 **	0.73 **	1						
11	Traditionalism	−0.22 **	0.10	−0.01	−0.01	0.03	−0.01	−0.15 *	−0.17 *	0.77 **	0.29 **	1					
12	AuthoritarianAggression	−0.18 *	−0.07	0.05	−0.16	−0.01	−0.06	−0.11	−0.14	0.69 **	0.42 **	0.21 **	1				
13	CriticalThinking	0.40 **	0.02	0.07	0.05	0.03	0.06	0.07	0.00	−0.13	−0.06	−0.20 **	0.01	1			
14	CriticalOpenness	0.41 **	3	0.01	0.04	0.03	0.03	0.02	−0.01	−0.13	−0.05	−0.22 **	0.04	0.92 **	1		
15	ReflectiveSkepticism	0.29 **	0.07	0.14	0.06	0.05	0.10	0.11	0.02	−0.13	−0.07	−0.14	−0.05	0.87 **	0.60 **	1	
16	Wisdom	0.53 **	0.11	0.08	0.21 *	0.17	0.20 *	0.16 *	0.15 *	−0.27 **	−0.14	−0.20 **	−0.24 **	0.34 **	0.37 **	0.23 **	1
17	Cognitive	0.42 **	0.09	0.10	0.20 *	0.25 **	0.24 **	0.14 *	0.15 *	−0.20 **	−0.17 *	−0.11	−0.18 *	0.25 **	0.27 **	0.15 *	0.80 **
18	Reflective	0.37 **	0.17 *	0.10	0.13	0.13	0.16 *	0.18 *	0.21 **	−0.08	0.00	−0.03	−0.15 *	0.24 **	0.25 **	0.19 *	0.78 **
19	Affective	0.42 **	−0.01	−0.03	0.18	0.00	0.08	0.04	−0.02	−0.35 **	−0.16 *	−0.35 **	−0.23 **	0.31 **	0.35 **	0.18 *	0.72 **
20	Creativity	0.22 **	0.13	0.04	0.13	0.14	0.13	−0.07	−0.03	−0.10	−0.05	−0.13	−0.03	0.37 **	0.38 **	0.27 **	0.29 **
21	NFC	0.44 **	0.06	0.15	0.18	0.26 **	0.24 **	0.10	0.15 *	−0.25 **	−0.16 *	−0.20 **	−0.18 *	0.42 **	0.43 **	0.31 **	0.53 **
22	OE	0.35 **	0.04	−0.09	0.16	0.06	0.08	−0.05	0.09	−0.45 **	−0.38 **	−0.37 **	−0.21 **	0.27 **	0.27 **	0.21 **	0.28 **
23	Imagination	0.14	−0.04	−0.19	0.07	−0.02	−0.01	−0.14	0.01	−0.15 *	−0.14	−0.13	−0.05	0.23 **	0.23 **	0.17 *	0.02
24	ArtisticInterests	0.27 **	0.02	−0.10	0.18	0.02	0.07	0.02	0.11	−0.25 **	−0.26 **	−0.11	−0.20 **	0.20 **	0.17 *	0.19 *	0.16 *
25	Emotionality	0.20 **	0.01	−0.08	0.07	−0.07	−0.04	−0.07	−0.02	−0.26 **	−0.24 **	−0.22 **	−0.10	0.02	0.02	0.00	−0.13
26	Adventurousness	0.15 *	0.08	−0.02	0.03	0.20 *	0.12	0.02	0.06	−0.06	−0.11	−0.02	−0.02	0.10	0.13	0.06	0.35 **
27	Intellect	0.32 **	0.06	0.05	0.11	0.02	0.07	−0.02	0.02	−0.22 **	−0.22 **	−0.12	−0.17 *	0.28 **	0.29 **	0.18 *	0.44 **
28	Liberalism	0.16 *	0.01	0.02	0.06	0.00	0.05	0.02	0.17 *	−0.63 **	−0.38 **	−0.68 **	−0.23 **	0.09	0.07	0.13	0.14
29	DOG	−0.40 **	0.08	0.08	−0.15	−0.11	−0.08	−0.19 *	−0.17 *	0.35 **	0.14	0.51 **	0.02	−0.16 *	−0.23 **	−0.05	−0.24 **
30	BIDR	0.23 **	0.11	0.03	0.06	0.01	0.03	−0.03	−0.10	0.17 *	0.17 *	0.12	0.10	0.15 *	0.12	0.16 *	0.21 **
31	SDE	0.20 **	0.07	0.21	0.05	0.02	0.08	−0.01	−0.08	0.14	0.12	0.12	0.07	0.11	0.10	0.09	0.29 **
32	IM	0.18 *	0.12	−0.14	0.05	0.01	−0.01	−0.04	−0.09	0.15 *	0.16 *	0.09	0.09	0.15 *	0.10	0.18 *	0.07
		**17**	**18**	**19**	**20**	**21**	**22**	**23**	**24**	**25**	**26**	**27**	**28**	**29**	**30**	**31**	**32**
17	Cognitive	1															
18	Reflective	0.43 **	1														
19	Affective	0.38 **	0.34 **	1													
20	Creativity	0.26 **	0.13	0.30 **	1												
21	NFC	0.50 **	0.31 **	0.42 **	0.43 **	1											
22	OE	0.30 **	0.05	0.32 **	0.34 **	0.39 **	1										
23	Imagination	0.10	−0.10	0.05	0.44 **	0.14	0.63 **	1									
24	ArtisticInterests	0.15 *	0.12	0.11	0.15 *	0.20 **	0.66 **	0.422 **	1								
25	Emotionality	−0.01	−0.37 **	0.11	0.04	−0.07	0.56 **	0.284 **	0.31 **	1							
26	Adventurousness	0.34 **	0.23 **	0.25 **	0.24 **	0.46 **	0.48 **	0.128	0.18 *	−0.04	1						
27	Intellect	0.35 **	0.30 **	0.37 **	0.23 **	0.46 **	0.63 **	0.190 **	0.27 **	0.09	0.34 **	1					
28	Liberalism	0.13	0.01	0.19 **	0.01	0.16 *	0.54 **	0.128	0.17 *	0.30 **	0.09	0.19 *	1				
29	DOG	−0.20 **	−0.11	−0.26 **	0.05	−0.14	−0.18 *	0.022	0.00	−0.09	−0.04	−0.11	−0.40 **	1			
30	BIDR	0.11	0.26 **	0.10	0.17 *	0.06	−0.02	0.036	0.09	−0.10	0.06	−0.06	−0.11	0.19 *	1		
31	SDE	0.14	0.42 **	0.10	0.16 *	0.07	−0.06	−0.061	0.04	−0.19 **	0.08	0.04	−0.12	0.21 **	0.82 **	1	
32	IM	0.05	0.04	0.06	0.13	0.05	0.02	0.106	0.11	0.00	0.04	−0.12	−0.07	0.12	0.85 **	0.39 **	1

* Correlation is significant at the 0.05 level (2-tailed). **. Correlation is significant at the 0.01 level (2-tailed). Note: SAT_ACT = ACT and SAT to ACT conversion; VSA = Very Short Authoritarianism (2018); NFC = Need for Cognition; OE = Openness to Experience; DOG = Dogmatism; AIAS = Adaptive Intelligence Attitudes Scale; BIDR = Balanced Inventory of Desirable Responding.

**Table 9:** Principal components matrix and rotated principal components matrix: Adaptive Intelligence Attitudes Scale (AIAS); psychometric assessments, Very Short Authoritarianism (VSA); Critical Thinking Dispositions Scale (CTDS); Wisdom (3D-WS-12); Creativity; Need for Cognition (NFC); Openness to Experience (OE); Dogmatism (DOG); and Balanced Inventory of Desirable Responding (BIDR).

	**Component Matrix**		
	**1**	**2**	**3**
AIAS	0.73	0.05	0.23
Letter Sets	0.36	−0.60	0.46
Figure Classification	0.36	−0.63	0.23
VSA	−0.53	0.36	0.42
CTDS	0.59	0.32	0.06
Wisdom	0.73	0.13	0.25
Creativity	0.54	0.49	−0.21
NFC	0.73	0.17	−0.04
OE	0.58	0.08	−0.53
DOG	−0.43	0.44	0.08
BIDR	0.13	0.52	0.62
	**Rotated Component Matrix**		
	**1**	**2**	**3**
AIAS	0.68	0.36	−0.01
Letter Sets	0.06	0.83	0.05
Figure Classification	0.03	0.75	−0.16
VSA	−0.25	−0.28	0.67
CTDS	0.67	0.01	0.00
Wisdom	0.71	0.31	0.04
Creativity	0.69	−.27	−0.15
NFC	0.72	0.13	−0.19
OE	0.50	−0.10	−0.60
DOG	−0.16	−0.47	0.37
BIDR	0.42	−0.04	0.70

Notes: Extraction method: principal component analysis. Rotation method: varimax with Kaiser normalization. Rotation converged in 6 iterations. The three principal components in the component matrix had eigenvalues greater than 1. Component 1 had an eigenvalue of 3.32, accounting for 30.16% of the variance in the data. Component 2 had an eigenvalue of 1.74, accounting for 15.79% of the variance in the data. Component 3 had an eigenvalue of 1.28, accounting for 11.61% of the variance in the data. Cumulative percent variance accounted for was 57.56%.

**Table 10:** Principal components matrix and rotated principal components matrix: Adaptive Intelligence Attitudes Scale (AIAS); cumulative GPA, ACT and SAT to ACT conversion (SAT_ACT); Very Short Authoritarianism (VSA); Critical Thinking Dispositions Scale (CTDS); Wisdom (3D-WS-12); Creativity; Need for Cognition (NFC); Openness to Experience (OE); Dogmatism (DOG); and Balanced Inventory of Desirable Responding (BIDR).

	**Component Matrix**		
	**1**	**2**	**3**
AIAS	0.75	−0.04	0.01
Cumulative GPA	0.19	0.63	−0.53
SAT_ACT	0.31	0.42	−0.67
VSA	−0.45	0.48	0.21
CTDS	0.62	0.02	0.23
Wisdom	0.73	0.09	0.05
Creativity	0.62	0.20	0.31
NFC	0.74	−0.01	0.09
OE	0.63	−0.25	−0.01
DOG	−0.40	0.46	0.38
BIDR	0.24	0.57	0.47
	**Rotated Component Matrix**		
	**1**	**2**	**3**
AIAS	0.70	−0.26	0.12
Cumulative GPA	0.05	0.13	0.83
SAT_ACT	0.10	−0.14	0.83
VSA	−0.28	0.63	0.04
CTDS	0.66	−0.05	−0.03
Wisdom	0.70	−0.13	0.18
Creativity	0.71	0.13	0.03
NFC	0.72	−0.19	0.09
OE	0.56	−0.40	−0.02
DOG	−0.19	0.69	−0.08
BIDR	0.46	0.63	0.06

Notes: Extraction method: principal component analysis. Rotation method: varimax with Kaiser normalization. Rotation converged in 4 iterations.The three principal components in the component matrix had eigenvalues greater than 1. Component 1 had an eigenvalue of 3.38, accounting for 30.77% of the variance in the data. Component 2 had an eigenvalue of 1.45, accounting for 13.18% of the variance in the data. Component 3 had an eigenvalue of 1.29, accounting for 11.76% of the variance in the data. Cumulative percent variance accounted for was 55.70%.

**Table 11:** Principal components matrix and rotated principal components matrix: Adaptive Intelligence Attitudes Scale (AIAS); psychometric assessments; cumulative GPA, ACT and SAT to ACT conversion (SAT_ACT); Very Short Authoritarianism (VSA); Critical Thinking Dispositions Scale (CTDS); Wisdom (3D-WS-12); Creativity; Need for Cognition (NFC); Openness to Experience (OE); Dogmatism (DOG), and Balanced Inventory of Desirable Responding (BIDR).

	**Component Matrix**			
	**1**	**2**	**3**	**4**
AIAS	0.75	0.07	−0.05	0.18
Letter Sets	0.31	−0.66	0.05	0.41
Figure Classification	0.29	−0.73	0.05	0.17
Cumulative GPA	0.19	−0.15	0.68	−0.42
SAT_ACT	0.36	−0.44	0.52	−0.38
VSA	−0.46	0.15	0.45	0.30
CTDS	0.61	0.23	−0.01	0.18
Wisdom	0.72	0.09	0.09	0.21
Creativity	0.58	0.42	0.14	−0.09
NFC	0.74	0.13	−0.02	−0.04
OE	0.61	0.20	−0.27	−0.40
DOG	−0.43	0.31	0.39	−0.03
BIDR	0.21	0.41	0.50	0.45
	**Rotated Component Matrix**			
	**1**	**2**	**3**	**4**
AIAS	0.73	0.22	−0.15	0.00
Letter Sets	0.10	0.83	0.02	0.05
Figure Classification	0.00	0.77	−0.14	0.20
Cumulative GPA	0.07	−0.03	0.11	0.83
SAT_ACT	0.10	0.28	−0.10	0.80
VSA	−0.25	−00.10	0.67	0.01
CTDS	0.67	0.05	−0.04	−0.05
Wisdom	0.73	0.22	−0.03	0.07
Creativity	0.67	−00.24	−0.04	0.16
NFC	0.70	0.06	−0.24	0.12
OE	0.51	−00.20	−0.58	0.12
DOG	−0.23	−0.39	0.46	0.13
BIDR	0.49	−0.07	0.65	0.03

Notes: Extraction method: principal component analysis. Rotation method: varimax with Kaiser normalization. Rotation converged in 6 iterations.The four principal components in the component matrix had eigenvalues greater than 1. Component 1 had an eigenvalue of 3.49, accounting for 26.82% of the variance in the data. Component 2 had an eigenvalue of 1.75, accounting for 13.50% of the variance in the data. Component 3 had an eigenvalue of 1.45, accounting for 11.13% of the variance in the data. Component 4 had an eigenvalue of 1.08, accounting for 8.34% of the variance in the data. Cumulative percent variance accounted for was 59.78%.

## Data Availability

Data are available on request from Arezoo Soleimani Dashtaki.

## Note

1 Factor analyses (with extraction method principal axis factoring and rotation method oblimin with Kaiser normalization) yielded very similar results. They are not shown to conserve space. They are freely available on request from the authors.

## Appendix A. Typical-Performance Items for Measuring Intelligence as an Attitude *

Please respond to each of the following statements by rating the extent to which the statement characterizes you, where 1 = never, 2 = rarely, 3 = sometimes, 4 = often, 5 = almost always or always.

1. When trying to solve a problem in my life, I consider how those affected by my solution might feel about the solution.**
2. Almost every time I encounter a new problem that interests me, I come up with new ideas for it.**
3. I am considered to be a person who often has different and even odd ideas.**
4. Although people talk a lot about ethics, in the end, what really matters is whether a solution to a life problem works [inverse scored]
5. I am generally open to new ideas and ways of doing things proposed by other people, even if the ideas originally seem off-base.**
6. When I have a life problem to solve, I often try to seek out an authority and ask them to solve it for me [inverse scored]**
7. I am quite willing to admit to mistakes, even though such admissions can be embarrassing.**
8. I am very concerned about coming up with solutions to life problems that allow me to fit in with my peers [inverse scored]**
9. When I have a creative idea, I expect I often will have to work hard to convince other people of its rightness or usefulness.
10. I am willing and often eager to try new ways of solving problems when old ones do not work.**
11. I can’t look out for everyone, so I have learned to look out only for those who really count in my life. [inverse scored]**
12. I do my best at everything I do. [Lie Scale item]
13. I don’t mind being a hypocrite because, when you get right down to it, so is everyone else. [inverse scored]**
14. When I make a life decision, I expect people to accept my decision without my having to go into details about why I made the right decision [inverse scored]
15. I find that I frequently lie to others—for their own sake—so that I don’t hurt their feelings. [inverse scored]**
16. When I think about solutions to problems, I ask where they may lead in the long-term, not just the short-term.**
17. I am aware of biases I have that may affect how I solve life problems.
18. I find that most of the criticism I receive is poorly thought through and just plain wrong. [inverse scored]**
19. I try to take into account the feelings of other people when I make life decisions.**
20. When it comes to how to conduct my life, I agree with my friends on practically everything [inverse scored]
21. I find that, when I have life problems, it is worthwhile to seek out advice from others.**
22. I have an intuitive sense of right and wrong that I can trust in virtually any situation [inverse scored]**
23. I have found that, when all is said and done, people who are fundamentally different from me have surprisingly little to offer to me when I need to solve life problems. [inverse scored]**
24. I find it frankly boring to listen to other people drone on about their views on issues [inverse scored]**
25. I have trouble letting go of ideas that I have believed for a long time (inverse scored).
26. When I solve life problems, I try to look at those problems from many different points of view.
27. I love finding new solutions for old problems.**
28. When I am in the process of solving a life problem, I try to keep track of whether my solution is working.**
29. I often ask questions that nobody else asks.**
30. I prefer following clear instructions in solving problems (inverse scored)**
31. I sometimes find myself asking whether the life problem I am trying to solve is actually the right problem for me to be working on.**
32. When it comes to life problems, I almost never make a mistake in my decisions [inverse scored]**
33. I try to apply common sense in my daily life, even when parts of me want to do otherwise.**
34. I usually avoid situations or problems that are new for me. (inverse scored)
35. I virtually never lie. [“lie” scale item]
36. The truth is that people do best when they put their own interests first [inverse scored]**
37. When I am done solving a problem, before putting the solution into effect, I try to make sure that the solution is actually the right one.**
38. I try to solve life problems quickly because, so often, “the who hesitate are lost.” [inverse scored]**
39. Whenever I make a mistake, I make sure I fix it. [Lie Scale item]
40. When there is social pressure to conform to what other people do, I conform because I know that, for better or worse, that is what gets people ahead. (inverse scored)**

* Lie Scale items were not used in the factor analyses because the distribution of scores was normal with no exceptionally high scores.

** Items retained for the revised tool after factor analysis.
